# RE-FB: An Efficient Vision Model for Feeding Behavior Detection and Group-Level Feeding Activity Assessment in Broilers Housed in Multi-Tier Cages

**DOI:** 10.3390/ani16213429

**Published:** 2026-10-30

**Authors:** Hongliang Guan, Tian Hua, Jialei Xue, Hao Bai, Yuliang Gao, Guobin Chang, Bin Li

**Affiliations:** 1 College of Information and Artificial Intelligence, Yangzhou University, Yangzhou 225009, China; msghl@outlook.com (H.G.); dx120230100@stu.yzu.edu.cn (J.X.); gyl@yzu.edu.cn (Y.G.); 2 College of Animal Science and Technology, Yangzhou University, Yangzhou 225009, China; huatian0202@163.com (T.H.); gbchang1975@yzu.edu.cn (G.C.); 3 Joint International Research Laboratory of Agriculture and Agri-Product Safety, The Ministry of Education of China, Yangzhou University, Yangzhou 225009, China; bhowen1027@yzu.edu.cn

**Keywords:** multi-tier-caged broilers, feeding behavior detection, feeding activity assessment, lightweight model

## Abstract

Automated monitoring of broiler feeding can improve management, but occlusion and scale variation hinder reliable detection in commercial multi-tier cages. This study developed a lightweight vision model for feeding behavior detection (RE-FB) and a feeding activity level (FAL) indicator for group-level assessment. The 2300 annotated images of yellow-feathered broilers were divided 8:1:1 for training, validation, and testing. RE-FB combines reparameterized multi-branch feature extraction, attention, and bidirectional multi-scale fusion to detect feeding birds and segment cages and feed troughs. On the test set, RE-FB-m achieved 89.7% precision, 87.3% recall, 92.1% box mAP@0.5, and 99.6% mask mAP@0.5, with 8.1 million parameters and a reported model inference speed of 158 FPS on the test platform. RE-FB-m achieved the highest box mAP@0.5 for feeding behavior detection among the evaluated Faster R-CNN, RT-DETR, and YOLO models. RE-FB was then applied to videos of four fixed groups monitored from 11 to 17 days of age. FAL was defined as the time-averaged proportion of the total birds in each monitored group detected as feeding, expressed as a percentage. Age-specific mean FAL values across the four groups ranged from 7.95% to 8.74%, with no significant age effect detected (*p* = 0.175). After controlling for group effects, the association between FAL and same-day weight gain was not statistically significant (partial *r* = −0.106, *p* = 0.614). RE-FB accurately and efficiently detects feeding behavior in caged broilers and generates group-level FAL records by aggregating detections over time. This approach provides a technical basis for non-contact monitoring of feeding activity in broiler production and can provide quantitative behavioral information for feeding management.

## Simple Summary

Monitoring feeding activity in stacked cages is difficult because birds often block one another from view. We developed RE-FB to detect feeding birds and locate cage and feed trough regions in camera images. On the test images, the model achieved 92.1% bounding box mAP at an overlap threshold of 0.5 and an inference speed of 158 frames per second on the test platform. Feeding detections were then aggregated over time to calculate the average proportion of birds detected as feeding in each group. Four fixed groups were monitored from 11 to 17 days of age. Feeding activity showed a consistent descriptive ranking among these groups, and its association with same-day weight gain was not statistically significant. The approach produced group-level feeding activity records. Studies with more flocks, longer observation periods, and concurrent intake and health measurements are needed to assess their use in feeding management.

## 1. Introduction

Yellow-feathered broilers are important indigenous premium meat chickens in China and occupy a substantial share of domestic poultry consumption. Their annual production was estimated to exceed 3.54 billion birds in 2025 [1]. Compared with white-feathered broilers, they have a longer production cycle and relatively lower feed conversion efficiency [2,3]. Feed commonly accounts for 60–70% of total production costs [4]. Real-time detection of feeding behavior and assessment of feeding activity can therefore provide an important basis for optimizing feeding strategies and improving management efficiency [5,6,7].

Assessing feeding activity in multi-tier-caged broilers requires continuous observation of feeding behavior and measurement of feeding duration. Conventional assessment relies mainly on manual observation, which is labor-intensive, time-consuming, and susceptible to observer subjectivity. It is therefore unsuitable for sustained monitoring on large farms and even less practical for flock-level activity assessment [8,9]. Sensor-based methods have consequently been explored in precision poultry farming. Radio frequency identification (RFID) has been used to monitor individual broiler visits to feeders and drinkers [10] and laying hen visits to functional areas [11]. Its principal advantage is that tag codes link observations to individual birds without requiring visual identification or illumination [11]. However, RFID requires tag attachment and handling of each monitored bird. Reading performance also depends on tag orientation, antenna placement, and interference from surrounding structures, including metal [10,11]. Extending monitoring across multiple cage tiers therefore requires adequate antenna coverage of the resources of interest and management of the tagged birds. Computer vision enables simultaneous observation of multiple birds without attaching devices to them, but detection alone does not establish persistent individual identity; estimating individual feeding time requires an additional tracking procedure [12]. Visual monitoring is also constrained by camera coverage, illumination, viewing angle, and occlusion by other birds or housing equipment [13]. In commercial multi-tier cages, camera placement must provide sufficient visibility of feeding areas, and expanding coverage requires consideration of image detail and processing capacity. Thus, RFID offers advantages for monitoring identified individuals at instrumented resources, whereas computer vision provides a non-contact option for observing feeding activity within visible cage areas.

Previous vision-based studies have addressed different components of poultry behavior monitoring. Guo et al. [13] compared convolutional classifiers, including DenseNet-264, using cropped images of broilers at 2, 9, 16, and 23 days of age from research pens. Feeding labels incorporated the bird’s position relative to the feeder and the location of its head above the feed. Their classification results highlight the relevance of age-related appearance changes, but classification of cropped birds does not itself locate feeding individuals within a complete cage scene. Fang et al. [14] combined DeepLabCut-based skeletal representations with a naive Bayesian classifier to distinguish six broiler behavioral states, including eating. This approach provides posture information for behavior analysis, whereas its use in densely occupied cages depends on the visibility of the required body keypoints.

Resource-oriented studies differ further in their behavioral definitions and outputs. Li et al. [15] combined image segmentation and linear regression to estimate bird numbers around a tube feeder and nipple drinkers in a floor pen containing 60 Ross 708 broilers aged 26–28 days. This approach characterizes resource occupancy rather than individual feeding duration. In four-tier cages housing Jinghong 1 laying hens, Hao et al. [16] used an improved Faster R-CNN with ResNet101 and PAN, labeling birds with their heads near or inside the trough as feeding. Their work demonstrates the feasibility of feeding detection in stacked cages, although the study population and labeling criterion differ from those of broiler feeding time studies. Nasiri et al. [12], for example, combined YOLOv3 head detection with distance-based tracking in a commercial-scale broiler house and estimated individual feeding time from head presence inside a feeder region. The present study adopts a comparable operational criterion, identifying feeding when a bird’s head enters the feeding area of the trough. However, detections are aggregated within fixed cage groups to calculate FAL, rather than used to reconstruct individual visits. The studies therefore share a head position-based definition but assess feeding activity at different levels.

Research on chicken identification and other behaviors, including feather pecking in cage-free hens, further illustrates the diversity of poultry monitoring tasks and settings [17,18]. For multi-tier-caged broilers, the relevant methodological challenge is to detect small or partly occluded feeding targets across cage tiers and associate those detections with the appropriate cage and feeder regions for group-level feeding activity assessment.

These studies provide a basis for visual poultry monitoring across several housing systems. Commercial multi-tier cages present additional challenges. First, suitable public datasets are scarce, which restricts training of robust models [19,20]. Second, mutual occlusion and substantial target-scale variation reduce detection accuracy, while large models with slow inference do not satisfy real-time requirements. Finally, limitations in model accuracy and commercial scene complexity have impeded development of a practical feeding activity assessment method for multi-tier systems.

This study aimed to develop and evaluate RE-FB for feeding behavior detection and group-level feeding activity assessment in broilers housed in multi-tier cages. The framework combines feeding bird detection with segmentation of cage and feed trough regions, allowing for feeding detections to be spatially associated with fixed cage groups and aggregated into FAL records.

## 2. Materials and Methods

### 2.1. Broiler Feeding Behavior Dataset

The performance of supervised deep learning models depends strongly on dataset quality. Public datasets matching commercial multi-tier broiler environments remain scarce because of production safety, regional policy, and copyright constraints. We therefore collected videos from multiple scenes, viewpoints, and cage tiers and used semi-automated annotation to construct a dataset for detecting broiler feeding behavior under commercial conditions.

For annotation, a bird was labeled as feeding when its head entered the feeding area of the trough. Proximity to the trough without head entry did not meet this criterion. The label represents a visually defined feeding position; it does not require confirmation of individual pecks or ingestion. This operational criterion is comparable to the head position criterion used by Nasiri et al. [12], although the housing and feeder configurations differ.

#### 2.1.1. Data Collection

Samples were collected at the Smart Farming Mobile Experimental Platform of the College of Animal Science and Technology, Yangzhou University (32 degrees 23′23.2″ N, 119 degrees 25′08.4″ E), and in the multi-tier poultry house at Liunan Beautiful Farm, Jiangsu Lihua Animal Husbandry Co., Ltd. (32 degrees 50′35.9″ N, 119 degrees 40′05.0″ E), Jiangsu, China. The mobile platform used three-tier A-frame cages, whereas the commercial house used four-tier H-frame cages, providing diverse viewpoints and cage levels.

Birds at both sites were male yellow-feathered broilers of the same breed. At the experimental platform, videos of birds aged 14–21 days were acquired from 12 to 18 July 2025. Two surveillance cameras (DS-2CD3T47EWD-L; Hikvision, Hangzhou, China) recorded at a resolution of 2560 × 1440 pixels and a frame rate of 25 FPS. The daily recording window was 08:00–20:00.

The commercial house dataset included birds at 14, 28, and 50 days of age and was collected during two acquisition periods. From 28 November to 5 December 2025, intermittent video clips were acquired using a Nikon Z5 digital camera (NIKON CORPORATION, Tokyo, Japan) at 1920 × 1080 pixels and 30 FPS. From 8 to 24 January 2026, four Hikvision DS-2CD3T47EWD-L cameras recorded at 2560 × 1440 pixels and 25 FPS, with a daily recording window of 08:00–20:00.

**Figure 1:**
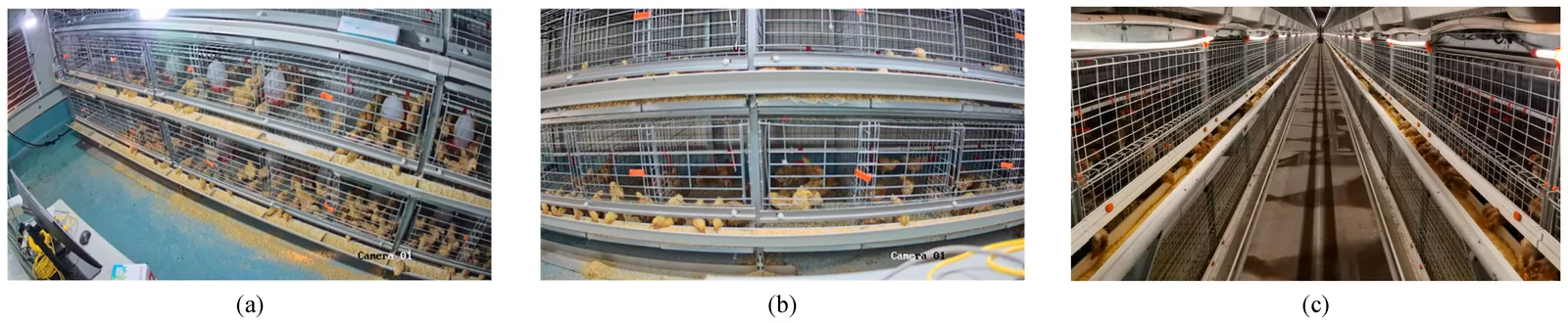
Representative acquisition views: (**a**,**b**) the experimental platform; and (**c**) the commercial multi-tier broiler house.

Cameras were aligned with the center of the monitored cage and directed downward at approximately 15–45°. The viewing angle was adjusted to provide a clear view of the feed trough region. The resulting camera view is shown in Figure 1. Recordings were acquired under the indoor lighting conditions present at each site.

Image selection involved two stages. First, frames were randomly sampled from each video recording, yielding 17,280 candidate images from the experimental platform, 4320 from the commercial house Nikon Z5 recordings, and 57,600 from the commercial house Hikvision recordings. The resulting candidate library contained 79,200 images. Second, the candidate images were manually screened to select 2300 images for annotation. Non-feeding birds within the selected images, including birds near the trough whose heads had not entered the feeding area, were retained as background. They were neither annotated as feeding targets nor labeled as a separate behavioral class. Images containing no feeding targets were excluded.

Feeding targets in the selected images were required to be sufficiently clear for annotation. Selection also considered background diversity, including variation in illumination and occlusion. The final dataset comprised 900 images from the experimental platform, 300 from the commercial house Nikon Z5 recordings, and 1100 from the commercial house Hikvision recordings. Tracing the sources of the 2300 selected images confirmed that no adjacent frames or near duplicates were included.

#### 2.1.2. Semi-Automated Image Annotation

Annotation quality directly affects supervised model performance [21]. Because feeding detections must later be spatially associated with their surroundings, the dataset included not only feeding individuals but also their cage compartments and corresponding feed troughs.

**Figure 2:**
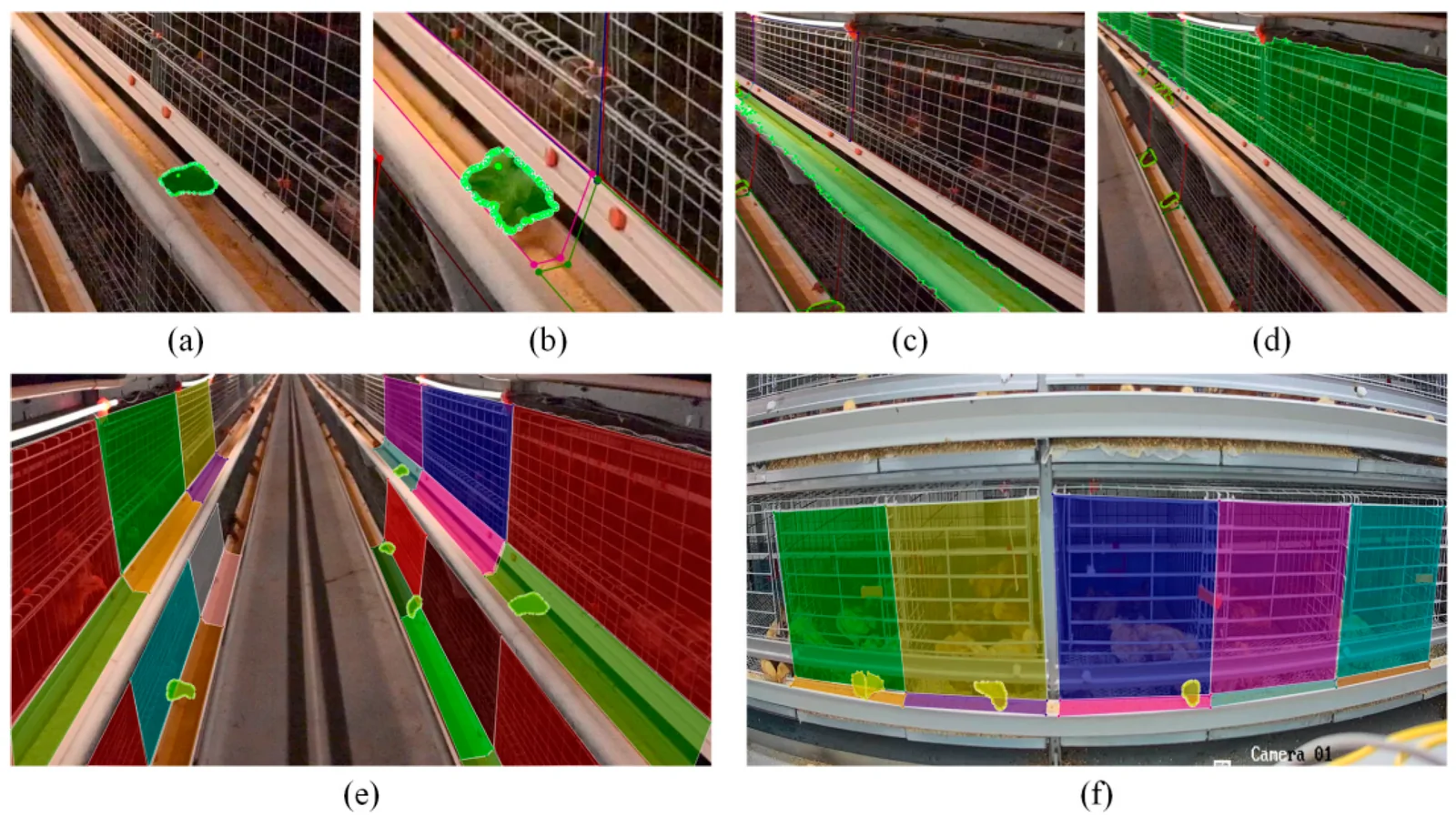
SAM 2-assisted annotation. (**a**) A target mask generated from click prompts; (**b**) instance confusion caused by occlusion; (**c**) incorrect feed trough boundary; (**d**) incorrect cage boundary; and (**e**,**f**) annotations after manual correction.

Segment Anything Model 2 (SAM 2) [22] was used to generate initial masks for the 2300 selected images. Click prompts identified target regions, as illustrated in Figure 2a. The annotated categories comprised feeding targets, cage compartments, and feed troughs. Feeding labels followed the head entry criterion defined in Section 2.1. Incorrect or missing regions, including confusion between adjacent targets and errors at trough and cage boundaries, were manually corrected and relabeled (Figure 2b–f).

Four annotators performed the annotation in Labelme version 6.3 using the Sam2 balanced model. A point prompt was placed on the head of each feeding bird to generate an initial mask outlining the head. The annotators manually inspected the masks and corrected incorrect or incomplete contours. When the model failed to delineate a head correctly, the mask was manually corrected, or the head was annotated manually. For partially occluded heads, instance boundaries followed the visible, unoccluded head contours. Approximate contours were annotated for blurred targets. The annotation protocol excluded severely blurred targets; none were present in the selected samples. A designated annotation quality reviewer inspected the annotations. Annotations that did not meet the quality requirements were returned for reannotation and reviewed again until accepted. Bounding boxes for all three classes were generated as the enclosing rectangles of the annotated instance masks.

Dataset partitioning was performed at the video level using one-hour source videos. All images from a given video were assigned to a single subset. The annotated images were allocated to training, validation, and test subsets at a ratio of 8:1:1. The validation subset was used during model development, and the test subset was used for the reported performance evaluation.

### 2.2. RE-FB Detection Model for Feeding Behavior in Multi-Tier-Caged Broilers

**Figure 3:**
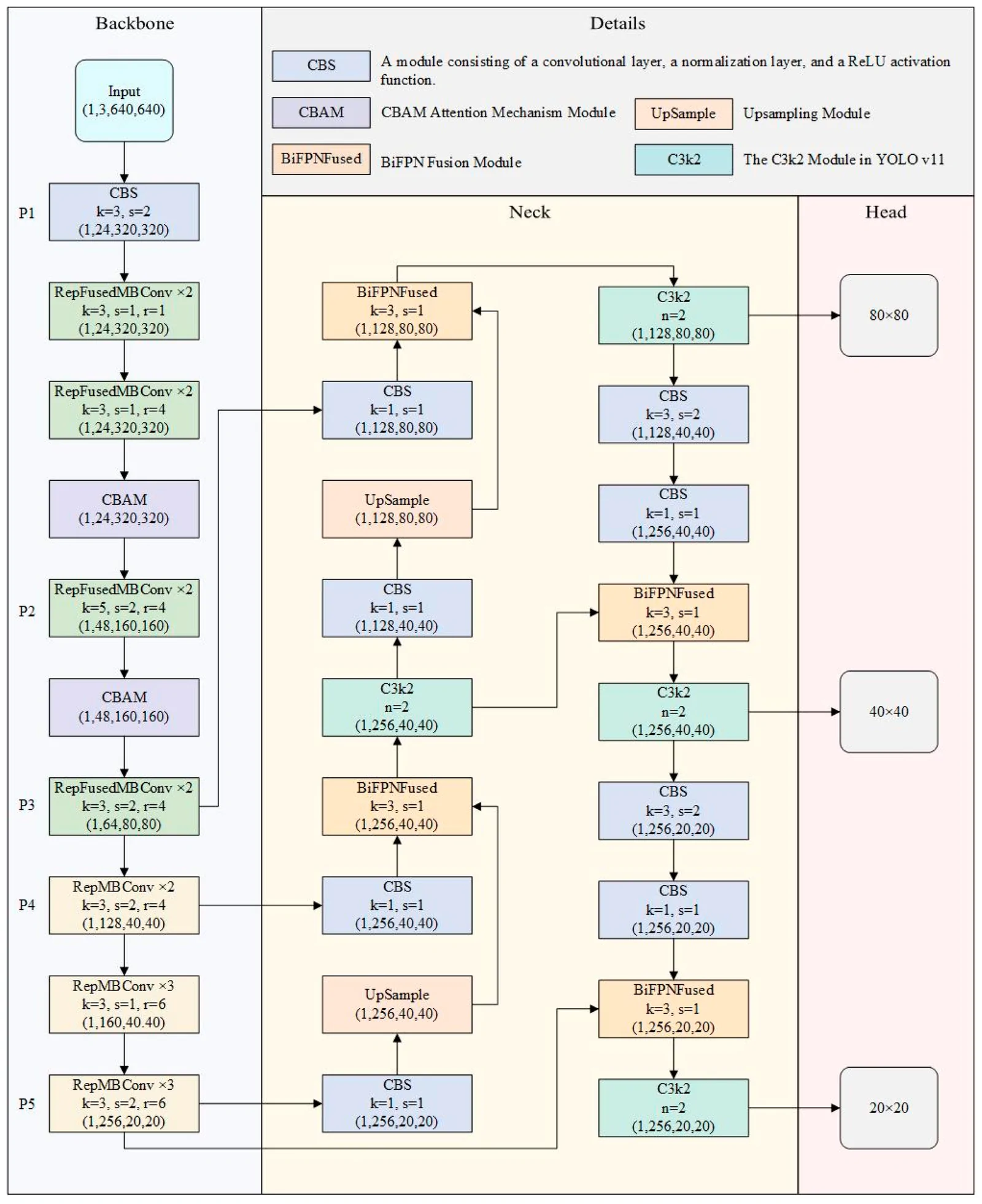
Architecture of the RE-FB feeding behavior detection model for multi-tier-caged broilers.

Feeding behavior detection provides the frame-level counts used in activity assessment. RE-FB comprises a backbone, neck, and prediction head (Figure 3). The model configuration contains 38 indexed module entries: 20 for the backbone, 17 for the neck, and one for the segmentation head. These entries describe the module arrangement rather than the total number of internal network layers.

The shallow backbone contains seven RepFusedMBConv blocks arranged into three stages. The first stage contains three blocks with 24 output channels and 3 × 3 kernels. The second stage contains two blocks with 48 output channels and 5 × 5 kernels. The third stage contains two blocks with 64 output channels and 3 × 3 kernels. The first block in the second and third stages uses stride 2, whereas the remaining blocks use stride 1. A CBAM module follows each stage, corresponding to configuration entries 4, 7, and 10.

The deeper backbone contains nine RepMBConv blocks arranged into three stages, each containing three blocks. Their output channel dimensions are 128, 160, and 256, respectively. All nine blocks use 3 × 3 kernels. The first blocks of the 128-channel and 256-channel stages use stride 2, and the remaining blocks use stride 1. The outputs of backbone entries 10, 16, and 19 provide features for the neck.

The neck contains four BiFPNFuse modules and four C3k2 modules. It first projects the deepest backbone feature to 256 channels and upsamples it by a factor of two using nearest neighbor interpolation. This feature is fused with the output of backbone entry 16 after a 1 × 1 convolution projects the latter to 256 channels. A C3k2 module processes the fused feature. A second projection and upsampling operation then produces a feature that is fused with backbone entry 10, with both inputs projected to 128 channels. The subsequent path uses two 3 × 3 convolutions with stride 2 to reduce spatial resolution and fuse the resulting features with entries 24 and 19, respectively. The unscaled E5 configuration specifies a repeat count of two for each of the four C3k2 entries.

The instance segmentation head (Segment) receives the outputs of entries 29, 33, and 36, with channel dimensions of 128, 256, and 256, respectively. These inputs represent three feature resolutions with a spatial resolution ratio of 4:2:1. The head is configured for three classes corresponding to feeding targets, cage compartments, and feed troughs. It predicts bounding boxes, class scores, and instance mask coefficients. The mask branch uses 32 mask prototypes and 256 intermediate channels in the prototype generation module. Instance masks are reconstructed by combining the shared prototypes with the coefficients predicted for each instance.

#### 2.2.1. Deep Semantic Feature Extraction with RepMBConv

Dense aggregation in multi-tier cages causes mutual occlusion and poorly defined target contours. Differences in viewing distance and cage tier also produce substantial target-scale variation. These conditions motivated the use of multi-scale features and computationally efficient feature extraction modules. The relationship between model scale and detection performance was evaluated separately in Section 3.2.

**Figure 4:**
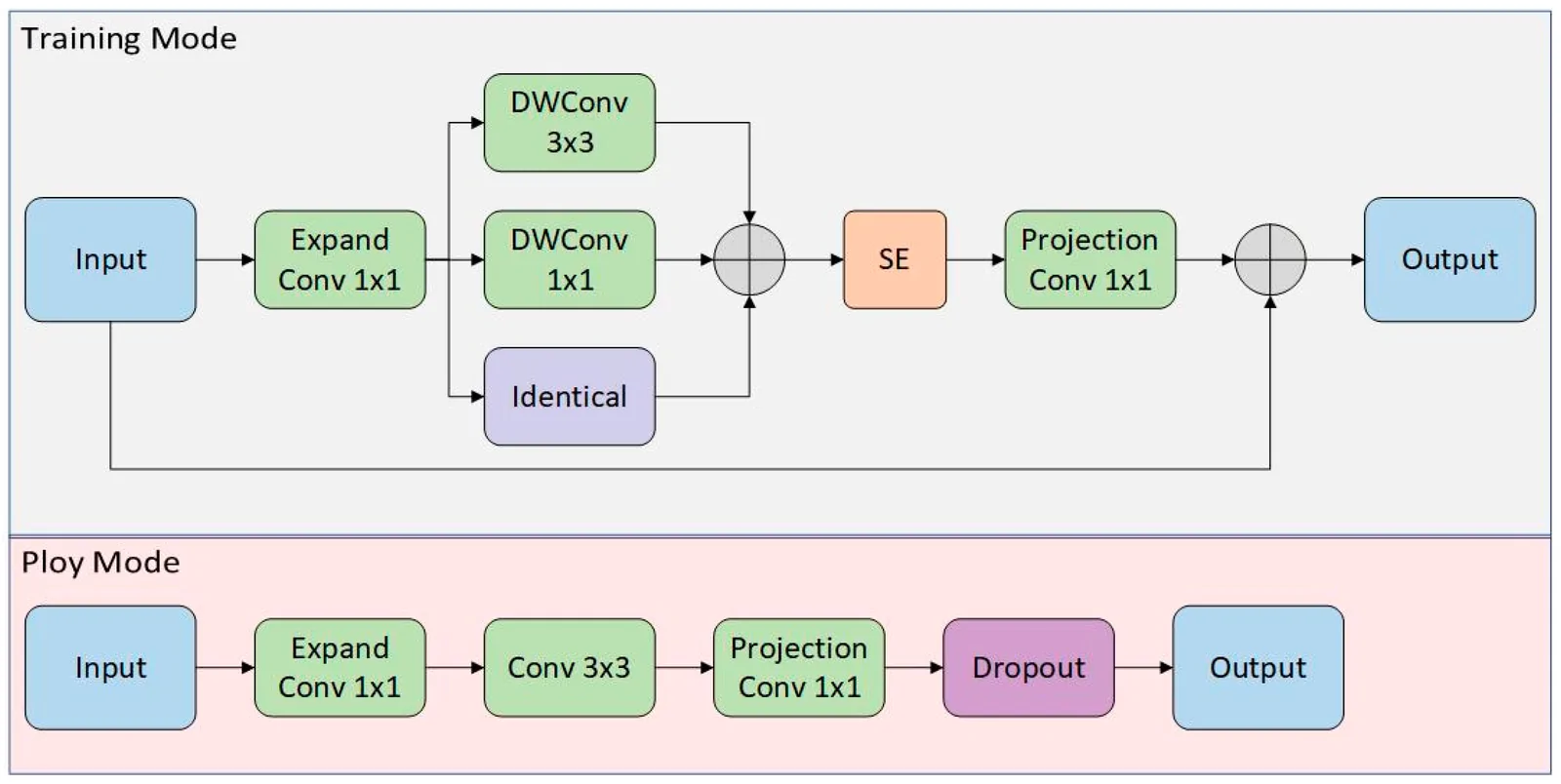
Architecture of the RepMBConv module.

We therefore designed a multi-branch reparameterized RepMBConv module for the RE-FB backbone. Inspired by the multi-branch training and single-branch inference strategy of RepViT [23], it redesigns the MBConv unit of EfficientNet [24] (Figure 4).

During training, RepMBConv uses parallel 3 × 3 depthwise convolution, 1 × 1 depthwise convolution, and identity branches. Their outputs are combined to represent local spatial features while retaining input information. At inference, the branches are structurally reparameterized into a single path. The effect of including this module was evaluated in the ablation experiments.

RepMBConv combines depthwise convolution with multi-branch training and structural reparameterization. It draws on the lightweight convolutional designs of MobileNet [25] and EfficientNet [24] and the separation of training and inference structures used in RepVGG [26]. These architectural connections motivate the design; its parameter count and detection performance are reported in Section 3.1.

#### 2.2.2. Shallow Semantic Feature Extraction with RepFusedMBConv

EfficientNetV2 noted that depthwise separable convolution can be inefficient in shallow layers because their high-resolution feature maps execute poorly on practical hardware; its lightweight advantage is more apparent at lower resolutions in deeper layers. EfficientNetV2 therefore introduced FusedMBConv, which removes the depthwise convolution component of MBConv [27].

**Figure 5:**
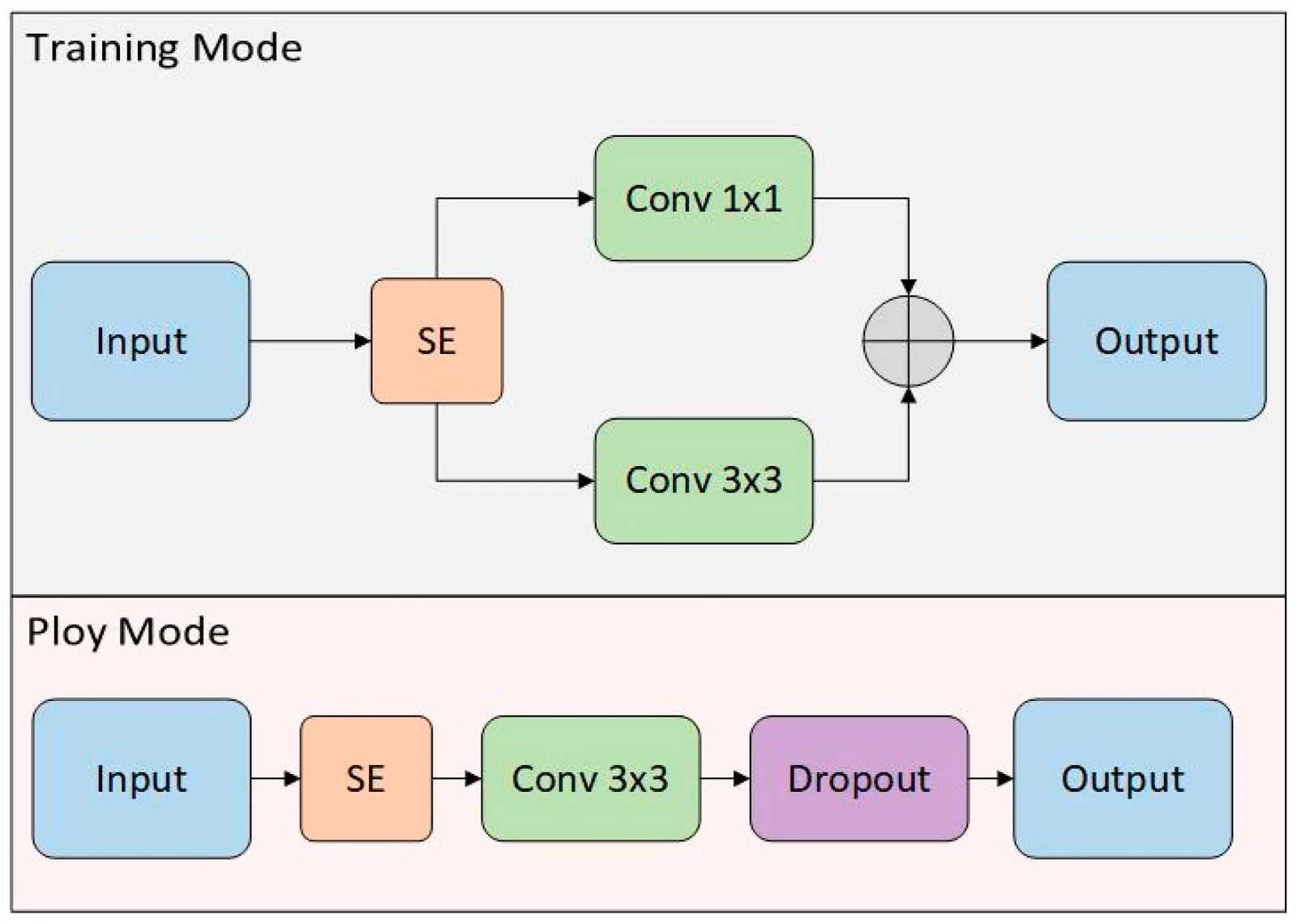
Architecture of the RepFusedMBConv module.

Following this principle, we adapted RepMBConv to create RepFusedMBConv for the shallow backbone (Figure 5).

The spatial convolution kernel size of RepFusedMBConv varies across the shallow backbone stages. The blocks with 24 and 64 output channels use 3 × 3 kernels, whereas those with 48 output channels use 5 × 5 kernels. The branch outputs are combined by element-wise addition. For inference, the 1 × 1 branch is converted and merged into the spatial convolution branch through structural reparameterization.

#### 2.2.3. CBAM and BiFPN

CBAM and a BiFPN neck were incorporated to provide channel and spatial attention and weighted multi-scale feature fusion, respectively [28,29].

CBAM sequentially applies channel and spatial attention to refine an intermediate feature map [28].

**Figure 6:**
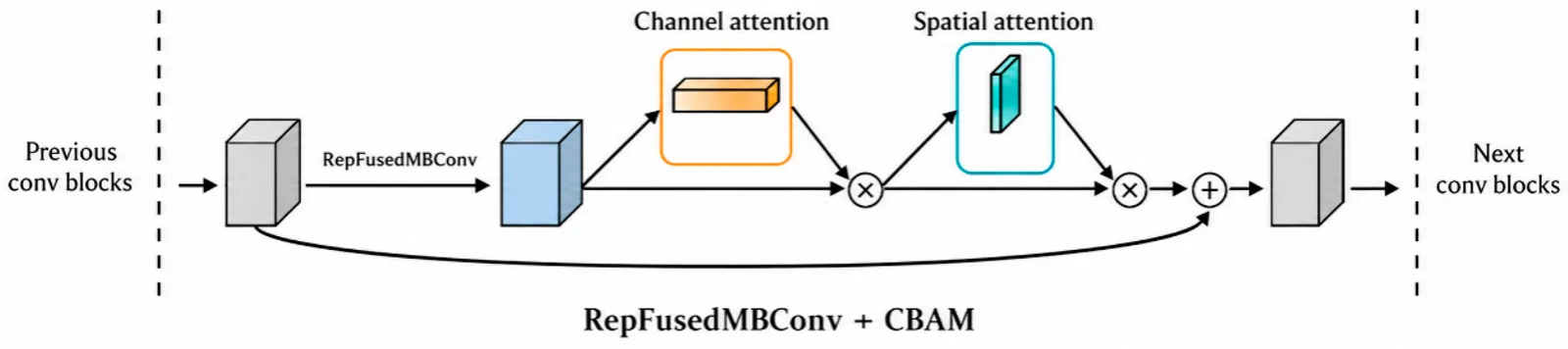
Workflow of CBAM following a RepFusedMBConv module.

CBAM was placed after RepFusedMBConv, as shown in Figure 6, to refine the shallow features before subsequent processing. Its contribution to the measured detection performance was examined by comparing configurations with and without this module.

The bidirectional feature pyramid network (BiFPN), introduced in EfficientDet, efficiently fuses multi-scale features through bidirectional information flow and learnable weights. To address scale variation, abundant small targets, and interference from feed-trough boundaries, BiFPN was applied to P3, P4, and P5 features in the RE-FB neck.

**Figure 7:**
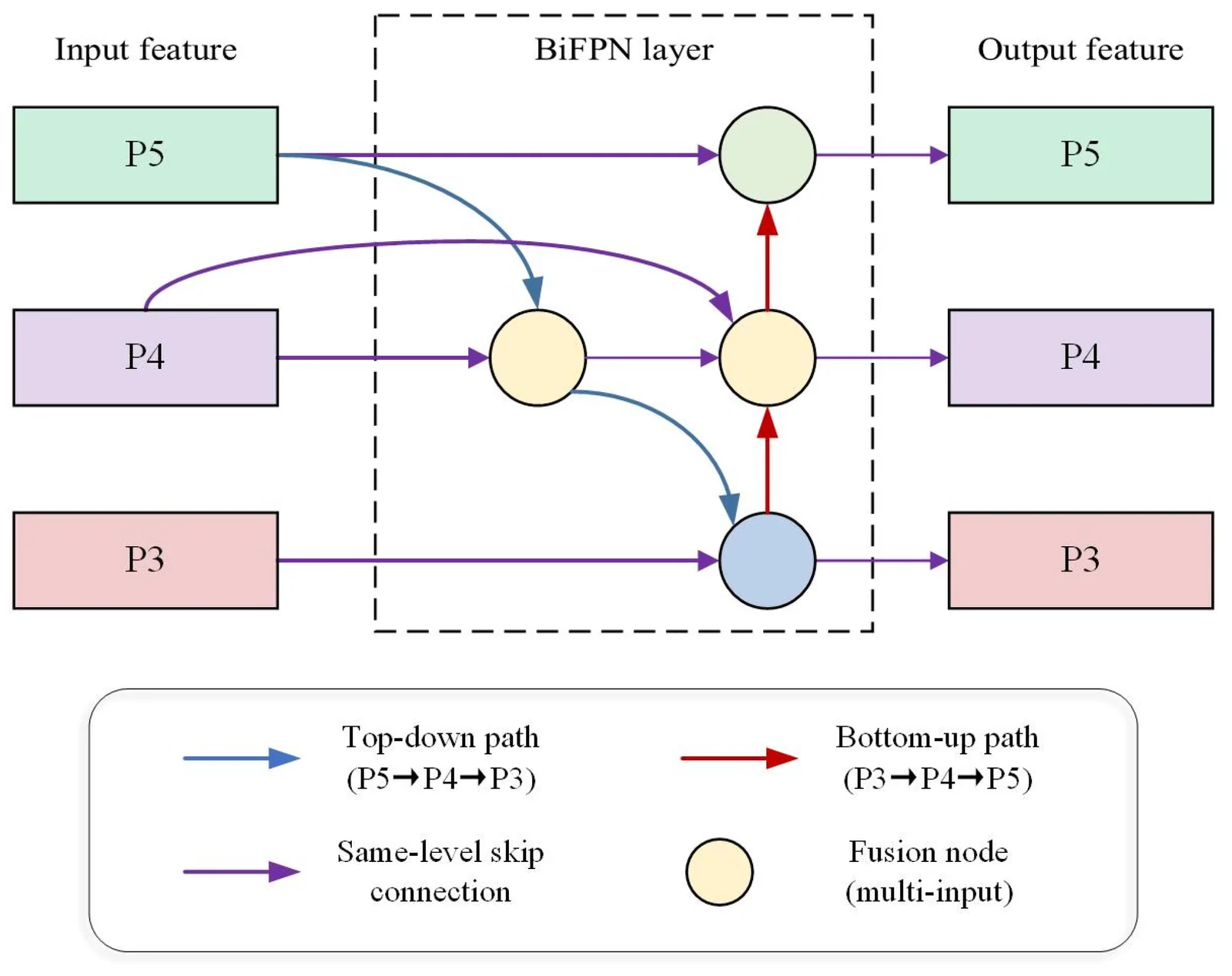
Bidirectional multi-scale feature fusion in BiFPN.

Figure 7 illustrates BiFPN fusion in RE-FB. The top-down path combines upsampled high-level features with higher-resolution features. The bottom-up path returns downsampled features to deeper levels. Learnable weights determine the relative contribution of each input to the fusion operation [29].

BiFPN replaces the original neck fusion structure with bidirectional weighted connections. The parameter count and detection results for this replacement are reported in the ablation study, where they can be assessed together with the subsequent backbone and attention changes.

### 2.3. Feeding Activity Assessment for Multi-Tier-Caged Broilers

**Figure 8:**
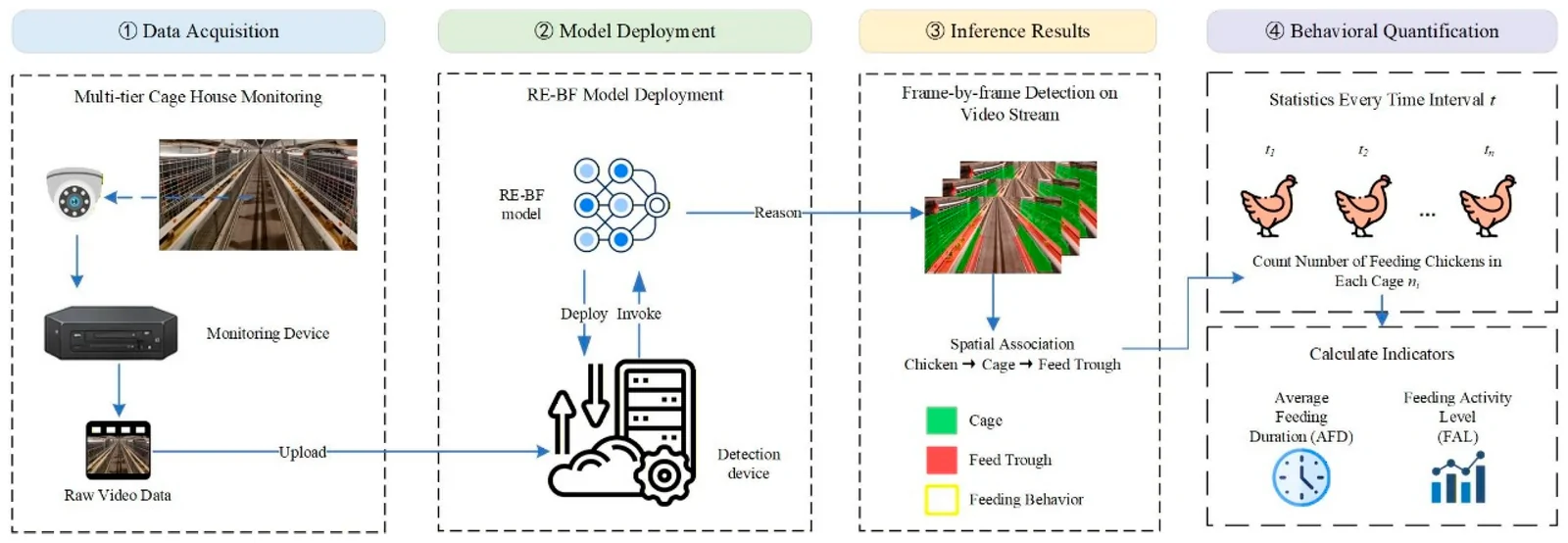
Workflow for assessing feeding activity in multi-tier-caged broilers.

To quantify flock feeding status over a defined period, we designed the workflow shown in Figure 8. A surveillance camera records the commercial multi-tier environment, video is uploaded to a cloud server running RE-FB, detections are saved, and time series analysis converts them into cage-specific feeding activity measures for comparison across ages and association with daily weight gain.

Feeding targets were assigned to cage groups through their spatial association with feed troughs. All target, cage, and trough regions were represented in the same image coordinate system. Each trough was first associated with the cage region having the highest intersection over union (IoU). When a trough and the candidate cage regions did not intersect, the cage with the smallest Euclidean distance between their region boundaries was selected.

Each feeding target satisfying the head entry criterion in Section 2.1 was then assigned to the trough with the highest positive IoU. Its cage group identity was obtained from the corresponding trough-to-cage association. When a target intersected more than one trough, the largest IoU determined its assignment. Equal IoU values were resolved using the distance between the target and trough centers. Each target was assigned to only one group. Partially visible targets were included when the visible head region satisfied the feeding criterion and could be associated with a trough using the same procedure.

Feeding activity level (FAL) was used as the primary behavioral measure, with average feeding duration (AFD) as an intermediate variable. For the FAL analysis, predictions were generated with a confidence threshold of 0.25. Class-specific non-maximum suppression used a bounding box IoU threshold of 0.50, with a maximum of 300 detections retained per image. Prediction time augmentation was disabled.

AFD was derived from the total feeding time represented by the frame-level feeding counts, divided by the number of birds in the monitored group. It therefore represents a group average estimate of feeding duration per bird, without reconstructing individual feeding histories. FAL normalizes AFD by the observation duration and expresses it as a percentage. With equally spaced sampled frames and a fixed group size, FAL is equivalent to the time-averaged proportion of all birds in the monitored group detected as feeding. Both measures depend on the specified head entry criterion and describe feeding activity rather than feed intake.

Let T denote the analyzed observation duration in seconds, *f_v_* the original video frame rate, and Δt the interval between sampled frames in seconds. The effective sampling frequency is *f*_s_ = 1 ÷ ∆t. At one sampled frame per second ∆t = 1 s and *f*_s_ = 1 Hz, irrespective of *f_v_*. For a complete observation period containing an integer number of sampling intervals, the sampled frame count *M* is

$$M=f_{s}\;T=\frac{T}{\Delta t}$$

M is the actual number of sampled frames analyzed. The equality assumes complete, equally spaced sampling over T.

Let *n*_i_ denote the number of feeding detections assigned to the monitored group in sampled frame *i*, and let *N* denote the total number of birds in that group. *N* was determined by on-site counting for each monitored group. It represents the group population, including birds not detected as feeding, rather than the number of visible birds or the number of distinct feeding birds. With equally spaced observations and fixed N, the group average feeding duration estimate AFD, in seconds per bird, is

$$\mathrm{AFD}=\frac{T}{M\;N}\sum_{i=1}^{M}n_{i}$$

FAL is AFD expressed as a percentage of T. Equivalently, it is the mean detected feeding count divided by the group population, expressed as a percentage:

$$\mathrm{FAL}=\frac{\mathrm{AFD}}{T}\times 100\%=\frac{1}{M\;N}\sum_{i=1}^{M}n_{i}\times 100\%$$

FAL expresses AFD as a percentage of the corresponding observation duration. Repeated FAL measurements were calculated for fixed cage groups to characterize temporal variation in feeding activity. A linear model with group included as a fixed effect was used to examine whether within-group variation in FAL was associated with same-day weight gain after adjustment for between-group differences. For the age comparisons, the Friedman test treated the four groups as blocks and the seven ages as repeated conditions. The 28 group day records represent repeated measurements of four groups, rather than 28 independent groups. Neither the Friedman test nor the group fixed effect adjustment explicitly models serial autocorrelation. Body weight was measured at 08:00 each day in the same 10 leg-banded birds selected from each cage. Their mean body weight was used to represent the cage. Mean daily weight gain was calculated as the difference between the mean body weights at 08:00 on the current and preceding days, corresponding to the 24 h interval ending at 08:00 on the current day.

### 2.4. Experimental Design

#### 2.4.1. Evaluation Metrics

Model performance was evaluated separately for feeding target detection and segmentation of environmental regions. Box precision (BP), box recall (BR), and box mAP@0.5 were calculated for feeding target bounding boxes. Mask mAP@0.5 was calculated for cage and feed trough instance masks. Parameter count (Params) and frames per second (FPS) described model complexity and inference speed, respectively. The corresponding formulas are provided in Table 1.

Feeding target boxes provide the counts used for FAL, while cage and feed trough masks define the environmental regions used for spatial assignment. The box, mask, and assignment tasks therefore require separate evaluation.

All models were evaluated using the same fixed test image list and corresponding ground truth annotations. Images were converted to RGB format and resized while preserving their aspect ratios using bilinear interpolation. Centered padding with a pixel value of 114 produced an input size of 640 × 640 pixels. Pixel values were scaled to the range from 0 to 1 before any additional normalization required by the individual model. Such normalization was retained from the corresponding training configuration. Additional internal resizing was disabled to maintain the specified input dimensions. Test augmentation was disabled. Evaluation used the same GPU platform described in Section 2.4.2, with a batch size of one and FP32 computation.

For detection performance evaluation, a confidence threshold of 0.25 was used to calculate box precision and recall. For AP calculation, predictions with confidence scores of at least 0.001 were retained to construct the precision–recall curves. The final prediction filtering used non-maximum suppression separately within each class, with a bounding box IoU threshold of 0.50. A maximum of 300 predictions was retained per image. These settings were applied consistently across the compared models.

Predictions were transformed back to the original image coordinates before evaluation. A detection was matched to an unmatched ground truth instance of the same class when their bounding box IoU was at least 0.50. Predictions were processed in descending confidence order, and the eligible ground truth instance with the highest IoU was selected. Unmatched predictions and unmatched ground truth instances were counted as false positives and false negatives, respectively. For mask evaluation, predicted masks were restored to the original image dimensions and binarized at 0.50. Matching used instance mask IoU instead of bounding box IoU.

Predictions from all models were converted to a common COCO representation. AP was calculated using the same pycocotools COCOeval implementation, with 101 recall thresholds, all object sizes, and a maximum detection setting of 300. Box mAP@0.5 was calculated for the feeding target class. Mask mAP@0.5 was calculated as the mean AP of the cage compartment and feed trough classes.

For feeding behavior detection, BP is the proportion of predicted feeding behavior bounding boxes that correctly match a ground truth box, whereas BR is the proportion of ground truth feeding behavior instances that are detected. Higher BP indicates fewer false positive feeding detections, and higher BR indicates fewer missed feeding birds.

Box mAP@0.5 evaluates feeding target detection against annotated bounding boxes at a bounding box intersection over union (IoU) threshold of 0.5. It does not directly quantify errors in feeding duration estimates or FAL and should not be directly compared with frame-level classification accuracy or feeding-time estimation accuracy reported in other studies.

Mask mAP@0.5 evaluates segmentation of the cage and feed trough classes at a mask IoU threshold of 0.5. Box mAP@0.5 and mask mAP@0.5 therefore evaluate different model outputs and should not be interpreted as interchangeable measures of the same task.

Params is the total number of trainable parameters. FPS is the number of images processed per second in the reported model inference benchmark. The latter describes model inference speed rather than the throughput or latency of the complete video processing workflow.

All models were evaluated on the same test subset using the same ground truth annotations and the same evaluation procedure for feeding behavior bounding boxes. Box mAP@0.5, calculated at a bounding box intersection over union (IoU) threshold of 0.5, was used to compare feeding behavior detection performance, with precision and recall reported as complementary metrics.

#### 2.4.2. Experimental Environment and Hyperparameters

The reported experimental platform used Ubuntu 22.04, Python 3.12, PyTorch 2.3.0, and CUDA 12.1. Hardware comprised one NVIDIA RTX 5090 GPU with 32 GB memory, an Intel Xeon Platinum 8470Q CPU, and 90 GB system memory. A 30 GB system disk and a 200 GB SSD data disk were used for software, data, and model outputs.

The saved configuration for the RE-FB model ablation run specified the Ultralytics segmentation task, a maximum of 200 epochs, early stopping patience of 50 epochs, a batch size of 8, and an input image size of 640 × 640 pixels. AdamW was selected, with an initial learning rate of 0.01, a final learning rate factor of 0.01, weight decay of 0.0005, and three warm-up epochs. Cosine learning rate scheduling was disabled. The run was configured without pretrained weights or checkpoint resumption. Automatic mixed precision, eight data loader workers, seed 42, deterministic execution, and device index 0 were specified. The nominal batch size was 64. The recorded loss weights were 7.5 for bounding box regression, 0.5 for classification, and 1.5 for distribution focal loss.

The RE-FB model training augmentation settings were 0.015 for hue variation, 0.7 for saturation variation, 0.4 for value variation, 0.1 for translation, and 0.5 for scale variation. Horizontal flipping had probability 0.5, and mosaic augmentation had probability 1.0, with mosaic closure configured for the final 10 epochs of the planned run. Rotation, shear, perspective transformation, vertical flipping, mixup, and copy/paste augmentation were disabled in the configuration. Multi-scale training was disabled, and the data fraction was 1.0. For mask targets, instance mask merging was enabled and the downsampling ratio was 4.

## 3. Results

### 3.1. Ablation Experiments

Five ablation experiments were conducted to evaluate the effects of RepMBConv, RepFusedMBConv, BiFPN, and CBAM (Table 2).

Table 2 reports detection metrics, parameter counts, and inference speeds for the five configurations. The E1 baseline achieved 82.9% box mAP@0.5 for feeding target detection.

Replacing the backbone feature extraction units with RepMBConv in E2 increased box mAP@0.5 from 82.9% to 88.5%, a difference of 5.6 percentage points. Parameter count decreased from 27.59 M to 16.89 M, while inference speed changed from 142 to 138 FPS.

Adding BiFPN in E3 reduced parameter count to 8.29 M, while box mAP@0.5 decreased to 79.0%. Thus, the lower parameter count in this configuration coincided with lower feeding target detection performance.

In E4, shallow RepMBConv units were replaced with RepFusedMBConv. Box mAP@0.5 increased to 87.9%, and inference speed reached 149 FPS.

Adding CBAM in E5 increased box mAP@0.5 to 91.0%, with BP of 90.0%, BR of 84.7%, and an inference speed of 153 FPS. Configuration E5 corresponds to RE-FB-l and incorporates RepMBConv, RepFusedMBConv, BiFPN, and CBAM.

**Figure 9:**
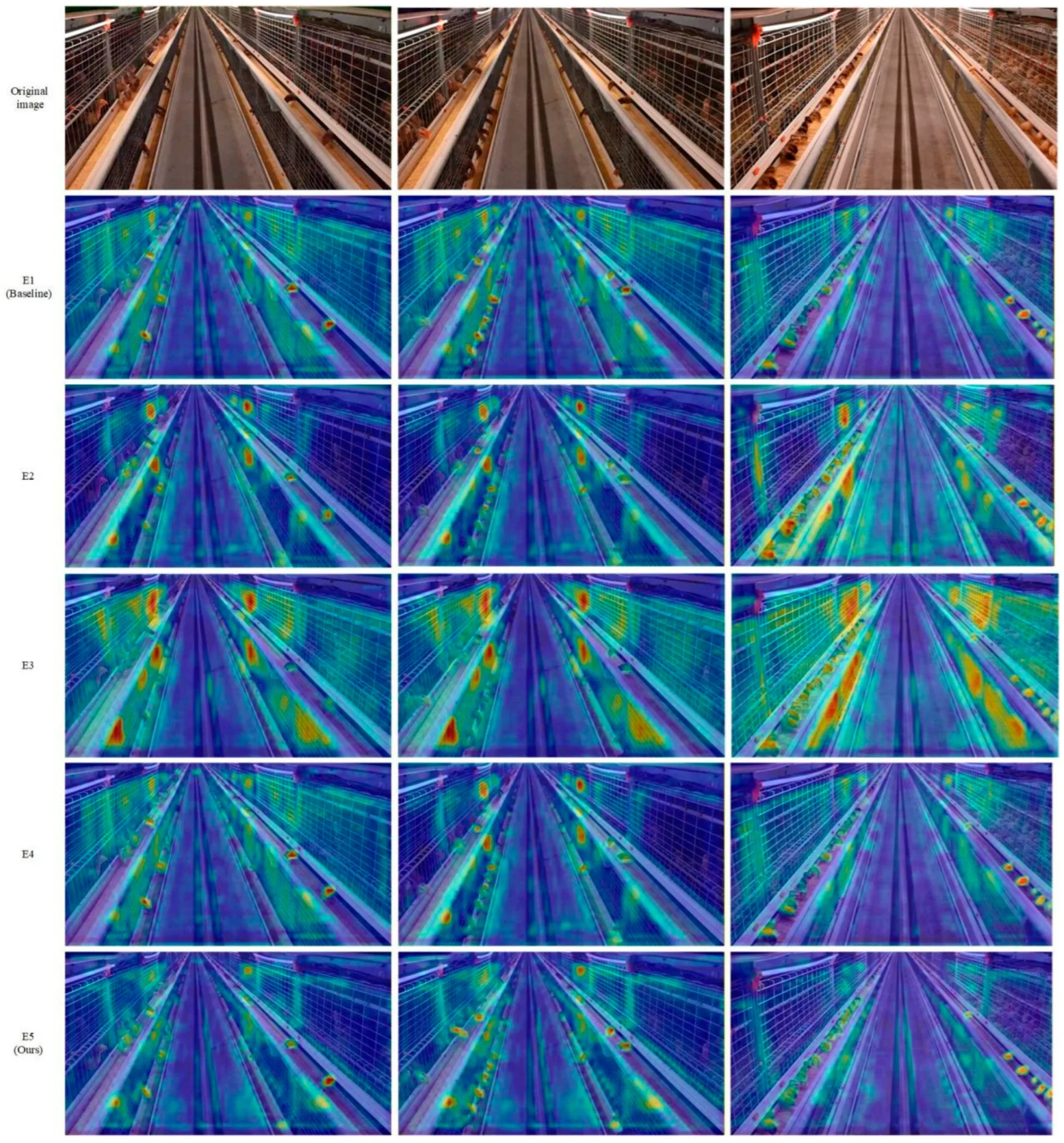
Attention heatmaps for models E1–E5. Warmer colors (red and yellow) indicate higher attention, whereas cooler colors (blue and purple) indicate lower attention.

**Figure 10:**
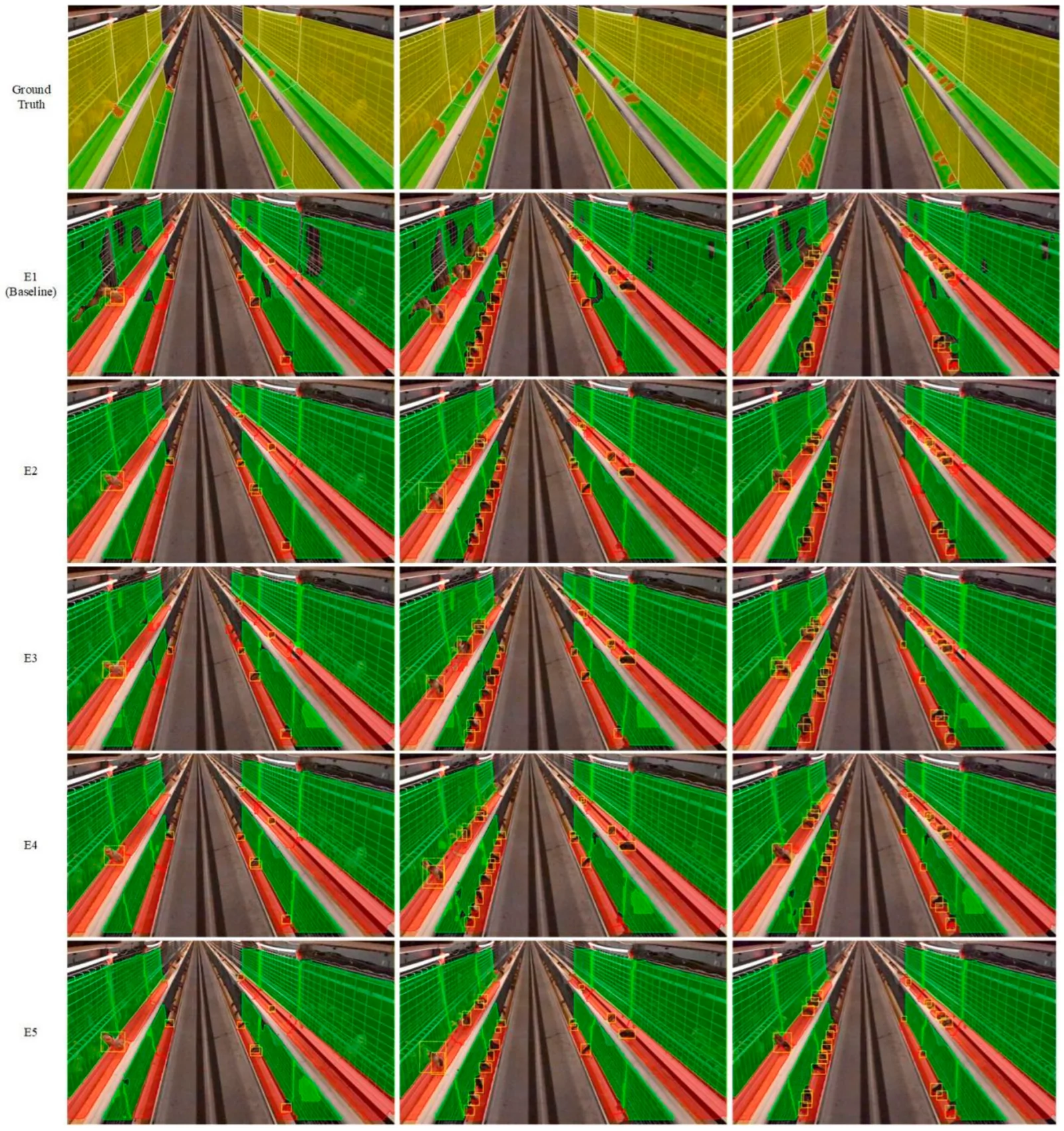
Example feeding target detections and cage and feed trough instance segmentation outputs for configurations E1 to E5.

Attention heatmaps and example detection and segmentation outputs for configurations E1 to E5 are shown in Figure 9 and Figure 10, respectively.

Figure 9 illustrates spatial responses over chicken heads, feed troughs, and cage structures in the displayed images. The distribution of responses varies among configurations. These heatmaps provide a qualitative view of model responses; quantitative comparisons of detection performance are given in Table 2.

Figure 10 illustrates feeding detections and the boundaries of cage and feed trough masks in selected images. The examples show differences in detection output and mask completeness. Quantitative model comparisons are reported in Table 2.

Among the ablation configurations, E5 had the highest feeding target box mAP@0.5. The mask mAP@0.5 values varied little across configurations. These comparisons describe the measured performance of the complete configurations.

### 3.2. Model Scaling

A compound depth-and-width scaling strategy analogous to the YOLO family was used to construct RE-FB-n, RE-FB-s, RE-FB-m, RE-FB-l, and RE-FB-x. Their accuracy and computational efficiency are compared in Table 3.

Mask mAP@0.5 for cage and feed trough segmentation ranged from 99.5% to 99.6% across the five scales. Feeding target box mAP@0.5 increased from RE-FB-n to RE-FB-m and was lower for RE-FB-l and RE-FB-x.

RE-FB-n contained 0.80 M parameters and achieved 82.9% box mAP@0.5 at 208 FPS. RE-FB-s contained 2.32 M parameters and achieved 88.7% box mAP@0.5 at 200 FPS.

RE-FB-m achieved the highest box mAP@0.5 among the five scales, at 92.1%, with BP of 89.7%, BR of 87.3%, 8.10 M parameters, and an inference speed of 158 FPS.

RE-FB-l and RE-FB-x contained 8.80 M and 19.31 M parameters, respectively, and achieved box mAP@0.5 values of 91.0% and 83.8%. RE-FB-x processed 121 frames per second. Increasing model size beyond RE-FB-m therefore did not improve the reported test set detection results.

RE-FB-m uses a depth multiplier of 0.5, a width multiplier of 1.0, and a maximum channel limit of 512. Compared with RE-FB-l, it achieved a higher feeding target box mAP@0.5, increasing from 91.0% to 92.1%, and higher recall, increasing from 84.7% to 87.3%. Its parameter count decreased from 8.80 M to 8.10 M, while inference speed increased from 153 to 158 FPS. RE-FB-m was therefore selected for the subsequent model comparisons, and its results are reported in the Abstract.

### 3.3. Comparison with Other Models

RE-FB-m was compared with the one-stage YOLO v8-m, YOLO v10-m, YOLO v11-m, and YOLO v12-m detectors, the two-stage Faster R-CNN, and the Transformer-based RT-DETR [30,31,32,33,34,35]. All models were trained from scratch without pretrained weights, using the same training and validation subsets and hardware environment. Training was limited to a maximum of 200 epochs, and the input image size was 640 × 640 pixels for all models. Shared training settings were kept consistent, while settings specific to each model followed the corresponding official training configuration. For each model, the checkpoint with the highest validation set mAP@0.5 was selected for test set evaluation. Evaluation followed the common procedure described in Section 2.4.1. The results in Table 4 compare the trained model configurations under these conditions.

RE-FB-m achieved the highest reported feeding-target BP (89.7%), BR (87.3%), and box mAP@0.5 (92.1%) among the evaluated models. Its box mAP@0.5 exceeded YOLO v11-m, RT-DETR, and Faster R-CNN by 5.4, 3.8, and 6.9 percentage points, respectively.

For the models with reported segmentation outputs, cage and feed trough mask mAP@0.5 ranged from 99.3% to 99.6%. RE-FB-m achieved 99.6%. These mask metrics evaluate region delineation.

RE-FB-m contained 8.1 M parameters, 63.8% fewer than YOLO v11-m, 74.2% fewer than RT-DETR, and 80.5% fewer than Faster R-CNN.

The reported model inference speed was highest for RE-FB-m at 158 FPS, compared with 42 FPS for Faster R-CNN and 68 FPS for RT-DETR.

### 3.4. Feeding Activity Assessment

Continuous video recordings of male yellow-feathered broilers monitored from 11 to 17 days of age were analyzed to assess the applicability of RE-FB to group-level feeding activity monitoring. Four fixed groups (ID = 01–04), containing 40, 40, 41, and 46 birds, respectively, served as the statistical units. All groups were maintained under the same environmental and management conditions. Feeding areas were located immediately outside the cages, and feed was supplied by an automated feeding system.

RE-FB identified feeding birds frame by frame, daily FAL was calculated, and concurrent daily weight gain was recorded to describe age-related changes and the association between FAL and weight gain.

**Figure 11:**
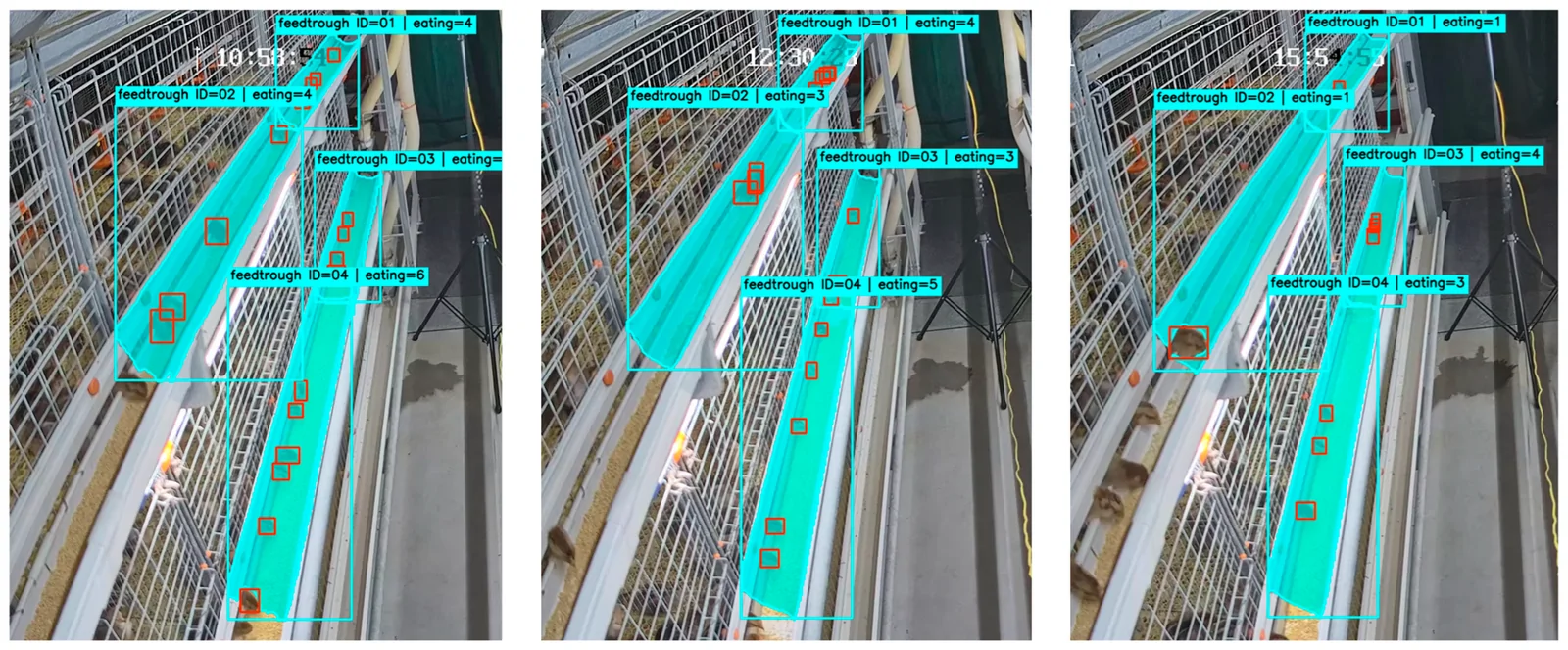
Representative RE-FB detections in the commercial poultry house. Red boxes indicate the heads of chickens that are feeding.

RE-FB produced feeding target detections and cage and feed trough masks. Feeding detections were assigned to four fixed groups, ID = 01 to ID = 04, using their spatial relationships with the cage and trough regions. Figure 11 shows representative detections with the environmental masks hidden for clarity.

Table 5 presents the FAL values of the four monitored units from 11 to 17 days of age. Mean FAL across the four units ranged from 7.95% to 8.74%. The Friedman test detected no significant differences in FAL among ages (χ^2^(6) = 8.97, *p* = 0.175). Descriptively, mean FAL therefore fluctuated within a relatively narrow range during the observation period. Because only four monitored units were included, this statistical result should be interpreted as exploratory rather than as evidence that age had no effect on feeding activity.

Mean FAL was 6.06 ± 0.22%, 7.69 ± 0.30%, 8.61 ± 0.41%, and 10.45 ± 0.60% for ID = 01, ID = 02, ID = 03, and ID = 04, respectively (mean ± SD, seven daily observations per group). The descriptive order ID = 04 > ID = 03 > ID = 02 > ID = 01 occurred at all seven ages, and within-group coefficients of variation ranged from 3.60% to 5.70%.

**Figure 12:**
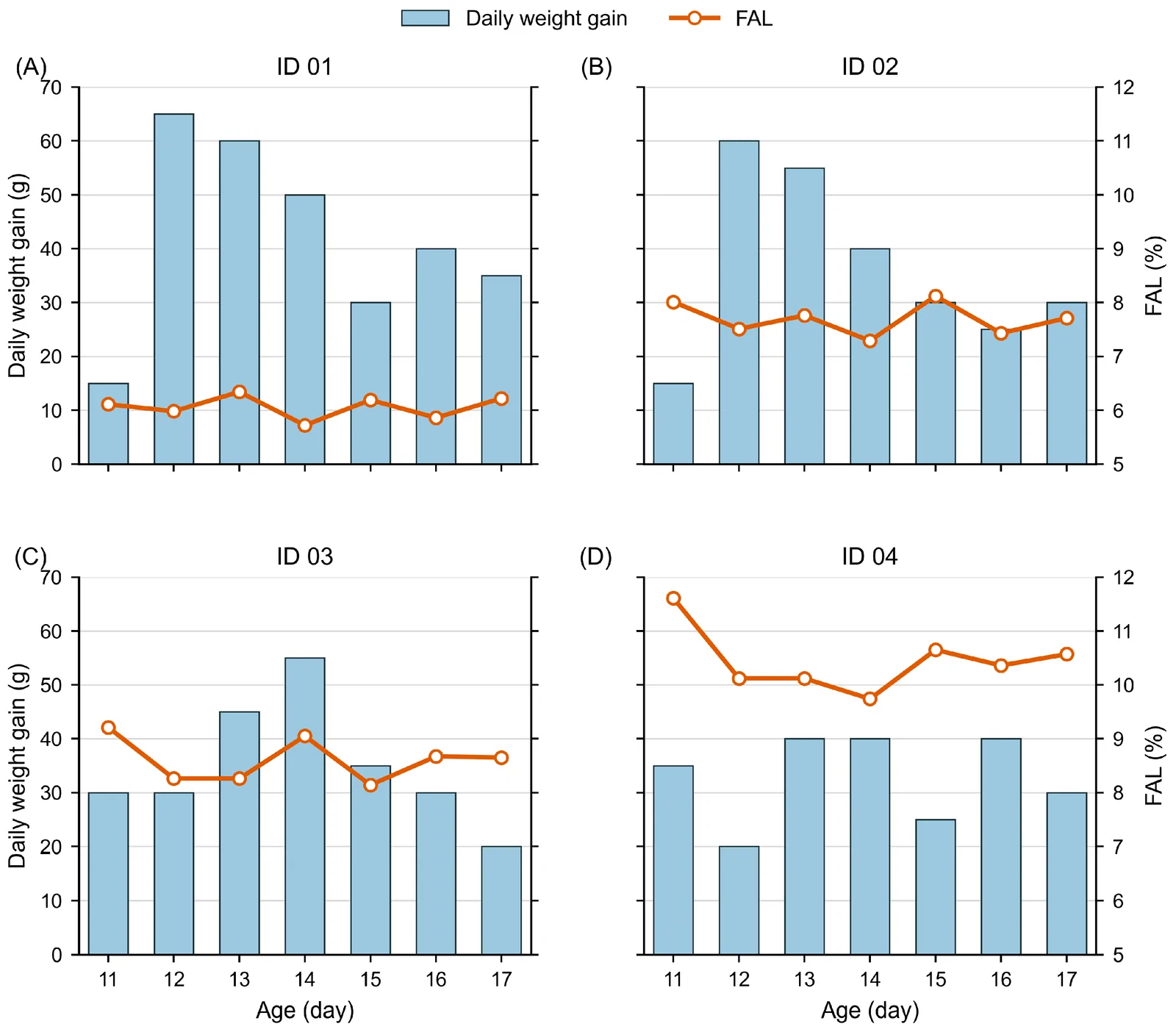
Changes in FAL and daily weight gain in four monitored groups from 11 to 17 days of age. (**A**) FAL and daily feed intake in cage ID = 01; (**B**) FAL and daily feed intake in cage ID = 02; (**C**) FAL and daily feed intake in cage ID = 03; (**D**) FAL and daily feed intake in cage ID = 04.

Figure 12 shows the changes in FAL and daily weight gain over this period. Daily weight gain averaged 36.61 ± 13.54 g, with a range of 15–65 g. Mean daily weight gain at 11–17 days of age was 23.75, 43.75, 50.00, 46.25, 30.00, 33.75, and 28.75 g, respectively. Daily weight gain increased from day 11 to a maximum on day 13, remained relatively high on day 14, decreased markedly on day 15, and subsequently remained below the levels observed on days 12–14. The Friedman test did not detect a significant difference in daily weight gain among ages (χ^2^(6) = 11.69, *p* = 0.069).

The association between FAL and same-day daily weight gain was examined using a linear model with group ID as a fixed effect. The group-adjusted association was weak and not statistically significant (partial r = −0.106, *p* = 0.614). This exploratory analysis comprised four fixed groups with seven daily observations each. Adjustment for group accounts for differences in group means but does not itself model temporal dependence among observations. Lagged relationships and associations with actual feed intake were not evaluated. The nonsignificant result does not establish the absence of a biological relationship between feeding activity and growth.

## 4. Discussion

RE-FB combines feeding target detection with cage and feed trough segmentation to produce group-level feeding activity records. The reported test results were 92.1% feeding target box mAP@0.5, 99.6% cage and feed trough mask mAP@0.5, and a model inference speed of 158 FPS. FAL was then calculated for four fixed groups observed from 11 to 17 days of age. Its group-adjusted association with same-day weight gain was not statistically significant. These results describe image-level model performance and an exploratory application of the resulting counts.

The model design draws on structural reparameterization, feature pyramid fusion, and attention. RepVGG [26] separates a multi-branch training structure from a simpler inference structure, while RepViT [23] examines efficient mobile convolutional designs. EfficientDet [29] uses weighted bidirectional fusion of features at different scales, and CBAM [28] refines features through channel and spatial attention. In the present ablation study, the BiFPN configuration reduced parameter count but also reduced box mAP@0.5; the subsequent RepFusedMBConv and CBAM configurations had higher detection scores. These results show that the outcome depended on the combination of components. The architectural studies provide a rationale for the design, whereas the attention maps in Figure 9 illustrate model responses without isolating the mechanism responsible for the performance differences. Direct numerical comparisons with those studies would also involve different datasets and tasks.

FAL summarizes the time-averaged proportion of birds in a group visually detected with their heads in the feeding area of the trough. It describes feeding participation under this visual criterion and does not measure the amount of feed consumed. Li et al. [15] estimated group feeding and drinking resource occupancy from images, whereas Nasiri et al. [12] tracked heads to estimate individual feeding time per visit. FAL retains the group-level spatial aggregation of the former approach and uses a head position criterion comparable to the latter. Its relatively narrow range across the seven observed ages describes the four monitored groups during this period. Detection performance was not reported separately by cage position or viewing condition. Camera perspective, visibility, and occlusion may therefore contribute to the persistent group ranking. The observed differences cannot be separated into behavioral and detection-related components from the present evaluation.

The group-adjusted association between FAL and same-day weight gain was not statistically significant. Feeding occupancy, time at a feeder, and feed consumption describe different aspects of feeding. Nasiri et al. [12] validated feeding time estimates against manual video observations, while Zuidhof et al. [3] measured body weight and feed intake to characterize growth and feed efficiency. These studies illustrate the additional measurements required to relate observed feeding activity to production outcomes. The present study did not measure feed intake, individual feeding duration, or feed efficiency, so it cannot determine how those quantities account for the observed FAL values or weight gains. Longer concurrent records are needed to examine temporal relationships, including possible lagged associations.

The methodological contribution is the aggregation of spatially assigned feeding detections into a repeated group-level measure. Its operational meaning follows from the head entry criterion and the denominator N. Whether FAL adds information to intake, body weight, or health measurements requires direct comparison with those outcomes.

Camera-derived behavioral records have potential management applications. Peña Fernández et al. [5] related broiler activity and occupation patterns to welfare outcomes, providing an example of validation against independently measured indicators. Prospective studies of FAL should include more flocks and independent experimental units, longer monitoring periods, and concurrent measurements of feeder access, feed intake, production, and health. Its use to detect unusual feeding patterns or inform management decisions depends on demonstrating those associations and evaluating detection thresholds against observed events.

The exploratory FAL analysis included four fixed groups over seven consecutive days, limiting statistical power, precision, and generalizability. The Friedman test’s chi-square approximation may be unreliable with so few groups, and the partial correlation *p*-value was not adjusted for serial dependence. The FAL application covered ages 11 to 17 days, whereas model development and evaluation data began at 14 days. The application at 11 to 13 days therefore extended beyond that range, and detection performance at these ages has not been independently established. Independent video-based validation of feeding counts and group assignments was not reported, leaving the effects of detection and assignment errors on FAL uncertain. Concurrent measurements of feed intake, individual feeding duration, feed efficiency, and health outcomes were unavailable, limiting assessment of biological relevance. Performance under farm, lighting, camera, and bird population conditions held out from model development remains unverified. The speed benchmark measured model inference, while throughput and latency of the complete monitoring workflow were not evaluated.

Future validation should include more independent groups and longer concurrent behavioral and production records to support the analysis of temporal dependence and lagged associations. Manually annotated video intervals are needed to quantify feeding count and group assignment errors and assess their effects on FAL estimates. External testing should use independent farms, production batches, camera layouts, and lighting conditions held out from model development. Evaluation of the complete workflow—from video transfer, decoding, and preprocessing to inference, spatial assignment, and FAL calculation—is also needed to establish sustained monitoring performance. Before operational thresholds for abnormal feeding activity are proposed, changes in FAL should be evaluated against independently recorded feeding, health, or management events.

## 5. Conclusions

RE-FB was developed for feeding target detection and group-level feeding activity assessment in broilers housed in multi-tier cages. RE-FB-m achieved 92.1% feeding target box mAP@0.5 and 99.6% cage and feed trough mask mAP@0.5, with 8.1 M parameters and a model inference speed of 158 FPS on the test platform. Spatially assigned feeding detections were aggregated into FAL records for four fixed groups observed from 11 to 17 days of age. Using FAL, this study explored the association between estimated group average feeding duration and daily weight gain, providing a methodological basis for research on feeding and growth. The group-adjusted association between FAL and same-day weight gain was not statistically significant. The FAL analysis was exploratory, covering four groups over seven days, with limited biological validation. Independent validation against measured feed intake and relevant production, health, and welfare outcomes is required before FAL is used to assess feeding efficiency, health, or welfare under commercial conditions.

## Figures and Tables

**Table 1:** Evaluation metrics for feeding behavior detection, cage and feed trough instance segmentation, model complexity, and inference efficiency.

Indicator Name	Formula	Variable Definitions
Box Precision (BP; feeding detection)	$\mathrm{BP}=\frac{{\mathrm{TP}}_{\mathrm{box}}}{{\mathrm{TP}}_{\mathrm{box}}+{\mathrm{FP}}_{\mathrm{box}}}$	*TP_box_* denotes the number of correctly predicted bounding boxes; *FP_box_* denotes the number of falsely predicted bounding boxes.
Box Recall (BR; feeding detection)	$\mathrm{BR}=\frac{{\mathrm{TP}}_{\mathrm{box}}}{{\mathrm{TP}}_{\mathrm{box}}+{\mathrm{FN}}_{\mathrm{box}}}$	*FN_box_* denotes the number of missed targets.
Average Precision (AP; feeding detection)	${\mathrm{AP}}_{c}=\int_{0}^{1}P_{c}(R_{c})dR_{c}$	*AP_c_* denotes the area under the Precision–Recall curve for the (*c*)-th class.
Box mAP@0.5 (feeding detection)	$\mathrm{Box}\mathrm{mAP}@0.5=\frac{1}{C}\sum_{c=1}^{C}{\mathrm{AP}}_{\mathrm{box},c}^{\mathrm{IoU}=0.5}$	*C* denotes the number of evaluated classes (one for feeding target box mAP@0.5); ${\mathrm{AP}}_{\mathrm{box},c}^{\mathrm{IoU}=0.5}$ denotes the average precision of the (*c*)-th class at a bounding box IoU threshold of 0.5.
Mask mAP@0.5 (cage/trough segmentation)	$\mathrm{Mask}\mathrm{mAP}@0.5=\frac{1}{C}\sum_{c=1}^{C}{\mathrm{AP}}_{\mathrm{mask},c}^{\mathrm{IoU}=0.5}$	${\mathrm{AP}}_{\mathrm{mask},c}^{\mathrm{IoU}=0.5}$ denotes the average precision of the (*c*)-th class at a mask IoU threshold of 0.5. The mean is taken over the cage compartment and feed trough classes.
Parameters	$\mathrm{Params}=\sum_{l=1}^{L}N_{l}$	*L* denotes the number of layers containing learnable parameters; *N_l_* denotes the number of learnable parameters in the (*l*)-th layer.
Frames Per Second	$\mathrm{FPS}=\frac{1}{t_{\mathrm{avg}}}$	*t_avg_* denotes the average inference time required to process a single image.

**Table 2:** Ablation results for the RE-FB model. BP, BR, and box mAP@0.5 evaluate feeding behavior detection, whereas mask mAP@0.5 evaluates cage and feed trough instance segmentation.

Exp.	RepMBConv	BiFPN	RepFused- MBConv	CBAM	BP (%)	BR (%)	Box mAP@0.5 (%)	Mask mAP@0.5 (%)	Params (M)	FPS
E1 (Baseline)					85.0	78.0	82.9	99.5	27.59	142
E2	√				86.9	81.2	88.5	99.5	16.89	138
E3	√	√			82.6	74.6	79.0	99.5	8.29	140
E4	√	√	√		85.8	85.7	87.9	99.5	8.79	149
E5 (RE-FB-l)	√	√	√	√	90.0	84.7	91.0	99.6	8.8	153

**Table 3:** Performance of RE-FB models at different scales. BP, BR, and box mAP@0.5 evaluate feeding behavior detection, whereas mask mAP@0.5 evaluates cage and feed trough instance segmentation.

Model	Depth	Width	Max Channel	BP (%)	BR (%)	Box mAP@0.5 (%)	Mask mAP@0.5 (%)	Params (M)	FPS
RE-FB-n	0.5	0.25	1024	83.8	78.3	82.9	99.5	0.80	208
RE-FB-s	0.5	0.5	1024	86.8	85.0	88.7	99.5	2.32	200
RE-FB-m	0.5	1.0	512	89.7	87.3	92.1	99.6	8.10	158
RE-FB-l	1.0	1.0	512	90.0	84.7	91.0	99.6	8.80	153
RE-FB-x	1.0	1.5	512	84.0	77.1	83.8	99.5	19.31	121

**Table 4:** Performance comparison of the evaluated models. BP, BR, and box mAP@0.5 evaluate feeding behavior detection, whereas mask mAP@0.5 evaluates cage and feed trough instance segmentation.

Model	BP (%)	BR (%)	Box mAP@0.5 (%)	Mask mAP@0.5 (%)	Params (M)	FPS
Faster R-CNN	83.4	79.6	85.2	—	41.5	42
RT-DETR	86.2	82.1	88.3	—	31.4	68
YOLO v8-m	83.1	80.5	84.4	99.3	25.9	142
YOLO v10-m	86.7	81.1	85.9	99.4	15.4	147
YOLO v11-m	86.0	82.6	86.7	99.5	22.4	151
YOLO v12-m	86.3	83.0	86.1	99.5	22.5	150
RE-FB-m (Ours)	89.7	87.3	92.1	99.6	8.1	158

**Table 5:** Feeding activity level (FAL, %) of four monitored groups from 11 to 17 days of age.

Age (Day)	ID = 01	ID = 02	ID = 03	ID = 04
11	6.11	8.01	9.21	11.61
12	5.98	7.51	8.26	10.12
13	6.34	7.76	8.26	10.12
14	5.72	7.29	9.05	9.74
15	6.19	8.12	8.14	10.65
16	5.86	7.43	8.67	10.36
17	6.22	7.71	8.65	10.57

## Data Availability

The data presented in this study are available on request from the corresponding author. The data are not publicly available because they are part of a proprietary dataset belonging to our collaborators.
